# Model Surfaces for Paper Fibers Prepared from Carboxymethyl Cellulose and Polycations

**DOI:** 10.3390/polym13030435

**Published:** 2021-01-29

**Authors:** Cassia Lux, Thomas Tilger, Ramsia Geisler, Olaf Soltwedel, Regine von Klitzing

**Affiliations:** Soft Matter at Interfaces, Department of Physics, Technical University of Darmstadt, Hochschulstraße 8, 64289 Darmstadt, Germany; lux@fkp.tu-darmstadt.de (C.L.); tilger@fkp.tu-darmstadt.de (T.T.); geisler@fkp.tu-darmstadt.de (R.G.); soltwedel@fkp.tu-darmstadt.de (O.S.)

**Keywords:** cellulose model surface, polyelectrolyte multilayers, dip-coating, carboxymethyl cellulose

## Abstract

For tailored functionalization of cellulose based papers, the interaction between paper fibers and functional additives must be understood. Planar cellulose surfaces represent a suitable model system for studying the binding of additives. In this work, polyelectrolyte multilayers (PEMs) are prepared by alternating dip-coating of the negatively charged cellulose derivate carboxymethyl cellulose and a polycation, either polydiallyldimethylammonium chloride (PDADMAC) or chitosan (CHI). The parameters varied during PEM formation are the concentrations (0.1–5 g/L) and pH (pH = 2–6) of the dipping solutions. Both PEM systems grow exponentially, revealing a high mobility of the polyelectrolytes (PEs). The pH-tunable charge density leads to PEMs with different surface topographies. Quartz crystal microbalance experiments with dissipation monitoring (QCM-D) reveal the pronounced viscoelastic properties of the PEMs. Ellipsometry and atomic force microscopy (AFM) measurements show that the strong and highly charged polycation PDADMAC leads to the formation of smooth PEMs. The weak polycation CHI forms cellulose model surfaces with higher film thicknesses and a tunable roughness. Both PEM systems exhibit a high water uptake when exposed to a humid environment, with the PDADMAC/carboxymethyl cellulose (CMC) PEMs resulting in a water uptake up to 60% and CHI/CMC up to 20%. The resulting PEMs are water-stable, but water swellable model surfaces with a controllable roughness and topography.

## 1. Introduction

Climate change has significantly increased the demand for recyclable and tunable materials with a natural origin in the last few years [1]. A material that shows great promise in replacing plastics is cellulose based paper. In its unmodified state, paper is highly biodegradable, biocompatible, and recyclable [2,3,4]. The cellulose fibers forming the network of paper are stabilized by hydrogen bonds and van der Waals forces. When the fibers get in contact with water, their swelling leads to a partial breakage of the inter-fiber stabilizing bonds [5]. To make paper (more) stable towards water, it is a common procedure to use functional additives in the paper making process, which link the fibers through more stable inter-fiber connections via chemical or physical bonding [6]. An established method for the modification is the use of polymer resins such as amine epichlorohydrins and urea formaldehyde. These resins lead to a homo- and hetero-crosslinking of the resin and the cellulose fibers [7,8]. The tuning of paper can not only lead to a higher wet strength, but can also modify characteristics such as flexibility and dry strength [9].

Planar cellulose model surfaces are a valid approach for investigating the functionalization of paper [10,11]. Model surfaces allow reducing measurement errors originating in the randomness of the fiber structure and open the possibility for many analysis methods for planar interfaces [11]. In addition, by tuning the surface structure of the thin film, both the chemical and physical factors of the interaction between the cellulose and a functional additive can be studied. Established methods for the preparation of model surfaces and thin films are spin-, dip-, and spray-coating and the Langmuir–Blodgett (LB) or Langmuir–Schäfer (LS) deposition methods [12]. A general challenge in studying paper chemistry using model surfaces based on cellulose is the preparation of the model surface itself. Cellulose is insoluble in water and in most organic solvents due to the complex structure of the fiber [13,14] and the hydrogen bonds of the linear high molecular weight polysaccharide [15]. Exceptions are two-component solvents, such as N,N-dimethylacetamide (DMA)/LiCl [16], and ionic liquids [17]. Successful dip- and spin-coating of cellulose containing thin films requires either a solution or a stable and homogeneous suspension prepared by dispersing cellulose colloids with dimensions in the size range of a few nanometers. Edgar et al. showed that cellulose nanocrystals in a stable aqueous suspension can be spin-coated onto a substrate, resulting in a homogeneous film with low roughness [18]. Using a cellulose solution with the aforementioned two-component solvents leads to the integration of a solid component into the thin film. Removing this component requires an additional washing step, which can alter the morphology of the model surface [12,19].

**Figure 1:**
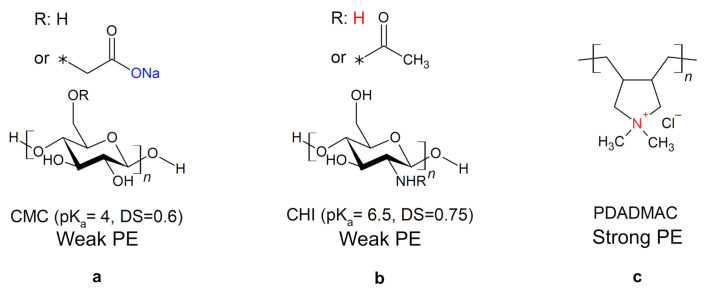
Molecular structures of the polyelectrolytes (PEs) used. The asterisk indicates on which position the functional group is attached to the polyelectrolyte, e.g., for CMC the asterisk represents the oxygen of the primary hydroxyl group. The negatively charged group of carboxymethyl cellulose (CMC) (**a**) is depicted in blue and the positively charged groups of chitosan (CHI) (**b**) and polydiallyldimethylammonium chloride (PDADMAC) (**c**) in red. For the weak PEs, the pK${}_{a}$-value and the degree of substitution (DS) are given.

In this work, cellulose model surfaces were prepared from an aqueous solution of carboxymethyl cellulose (CMC) (Figure 1a) by dip-coating. Compared to cellulose, in CMC, the primary hydroxy groups at the C6-atoms are partly substituted with a carboxymethyl group. The negative charge of the carboxy group enables the formation of polyelectrolyte multilayers (PEMs) by alternating the dip-coating of CMC and a polycation, chitosan (CHI) (Figure 1b) or polydiallyldimethylammonium chloride (PDADMAC) (Figure 1c). The molecular structure of the biopolymer CHI is similar to that of cellulose, as it is a β-1,4 linked polyamino-saccharide. It can be used to tune both the wet and dry strength of paper through physical linkage of the fiber [20]. PDADMAC, on the other hand, is a strong synthetic polyelectrolyte (PE). The charge density of the weak PEs CMC (pK${}_{a}\approx 4$) and CHI (pK${}_{a}\approx 6.5$) is pH dependent, while PDADMAC has a permanent positive charge.

The goal of this work is to determine the influence of the chemical composition (PDADMAC vs. CHI) and preparation condition (pH, PE concentrations, CMC vs. polycation as the outermost layer) on the morphology and swellability of the resulting PEMs. Besides the chemical structure, PDADMAC and CHI differ in their mobility and charge density. The system CHI/CMC prepared by dip-coating was partly the subject of works by the groups of Beppu et al. [21,22,23] and Lui et al. [24]. Beppu et al. determined the influence of the pH-value, salt concentration, and molecular weight on the formation of CMC/CHI PEMs. Contrary to their work, the prepared PEMs in this study are mainly terminated with the CMC layer. In addition to this, the outcome of the CHI/CMC PEMs are compared to the PDADMAC/CMC PEMs, and the experimental work is extended by analytical methods such as quartz crystal microbalance with dissipation monitoring (QCM-D) and ellipsometry to study the deposition kinetics and the swellability of the PEMs. Although the preparation of PEMs based on CMC has been studied in the literature, the preparation of cellulose model surfaces built from weak and strong polyelectrolytes and the tunability of the surface topography are still lacking. The topography of the PEMs is visualized by AFM.

## 2. Materials and Methods

### 2.1. Materials

The PEs sodium carboxymethyl cellulose (CMC, M_W_ = 250 kDa, degree of substitution (DS) = 0.6), polyethylenimine (PEI, M_W_ = 750 kDa, 50 wt-% in H_2_O), and chitosan (CHI, M_W_ = 50–190 kDa, DS = 0.75) were purchased from Sigma Aldrich (Darmstadt, Germany). Linear polydiallyldimethylammonium chloride (PDADMAC, M_W_ = 150 kDa) was purchased from PSS (Mainz, Germany). The ammonia solution (25%), hydrochloric acid (1 mol/L), and sodium hydroxide solution (1 mol/L) were purchased from Merck KGaA (Darmstadt, Germany). Sulfuric acid (100%) and hydrogen peroxide (30%) were purchased from Carl Roth (Karlsruhe, Germany) and the glacial acetic acid from VWR (Darmstadt, Germany). Ultrapure water was obtained from a Milli-Q-system from Merck with a resistance of 18 MΩ cm.

### 2.2. Preparation of PEMs

The PEMs were prepared by dip-coating. As the precursor, polyethylenimine was used at a concentration of $10^{-2}$ monoM (monoM refers to the concentration of monomer). The other PEs were dissolved in ultrapure water at the intended concentration of 1 g/L, unless stated otherwise. For a better solubility of the polycation, glacial acetic acid (HAc) of 0.1 M was added to the CHI solution. For varying CHI concentrations, the amount of HAc was adapted, so that the ratio of CHI and HAc remained constant (17.2 equiv. of HAc). Using hydrochloric acid and sodium hydroxide solutions (0.1 or 1 M), the pH value of all PE solutions (excluding PEI) and the rinsing water was adjusted to 4, unless stated otherwise.

Double side polished silicon wafers from Siegert Wafer (Aachen, Germany) were etched in piranha solution (3:1 with H_2_SO_4_:H_2_O_2_) for 30 min and stored in ultrapure water until use, but at most for 2 h. Using a layer-by-layer dip robot (Riegler & Kirstein, Berlin, Germany), the PEMs were prepared by first adsorbing the precursor PEI for 30 min, followed by 10 min of adsorption of the polyanion CMC and 10 min of adsorption of the polycation CHI or PDADMAC. After each adsorption step, the layers were rinsed by dipping three times into rinsing water for 2 min, 1 min, and 1 min. Through the alternation of the subsequent adsorption of the polyanion and polycation, the PEM forms with the desired number of bilayers (NoBL). In the present work, the PEI/CMC layer is defined as the first bilayer; therefore, the top layer in a bilayer is CMC.

### 2.3. Ellipsometry

The measurement of the thickness and refractive index was carried out using a Null-Ellipsometer (Optrel, Sinzing, Germany) with a PCSA setup (polarizer-compensator-sample-analyzer). The wavelength of the laser was 632.8 nm, and the angle of incidence was set to 70${}^{\circ }$. The measurements for determining the swelling ratio of the PEMs (Section 3.5) were carried out in a home-built humidity cell, which was placed in the beam path and connected to a nitrogen flow. By partly directing the nitrogen flow through two washing bottles filled with water at 25 ${}^{\circ }C$, the humidity inside the cell was controlled. The relative humidity (RH) and the temperature were measured by a thermohygrometer (Rotronic, Ettlingen, Germany). The ratio between dried and saturated nitrogen was adjusted to reach values of the relative humidity between 0% and 95% RH, with at least 10 steps for each measurement. After reaching a constant reading of the humidity, the PEM was left to equilibrate until constant ellipsometric angles were reached. When preparing a PEM on a silicon wafer and measuring at ambient conditions, a two-layer model is required, as described in Table 1. The SiO_x_ is a thin oxide layer on the substrate. The thickness of the oxide layer is an average value, which was determined by Löhmann et al. [25] using X-ray reflectometry. The depicted errors for the measurements at ambient conditions are the calculated standard deviation of the values of thickness and refractive index on 5 different spots on the samples. For the swelling measurements, a propagation of uncertainty dependent on the deviation of the measured thickness and refractive index was calculated.

### 2.4. AFM

Atomic force microscopy (AFM) was used to characterize the topography of the PEMs. The AFM measurements were carried out at ambient conditions (≈40% RH) with the MFP-3D SA (Asylum Research, Oxford Instruments, Scotts Valley, CA, USA). The cantilevers used were the AC160TS-R3 with a silicon probe and a tip diameter of 7–8 nm, also from Asylum Research. To determine the roughness of the PEMs, images of 25 µm^2^ were taken at three different positions of the sample, and the roughness was determined from nine randomly selected 1 µm^2^ areas of the image. The roughness is defined as the root mean square (RMS) of height deviations of the surface mean plane. The depicted error bars correspond to the standard deviation of the 27 spots measured. For PEMs with surface patterns with a correlation length in the µm range, the roughness was determined on the whole 25 µm^2^ area. In all cases, the two different roughness measurements (1 µm^2^, 25 µm^2^) led to similar values.

### 2.5. QCM-D

The adsorption kinetics of the PEM formation process were measured in situ by QCM-D measurements (QCM with dissipation monitoring, Q-Sense Explorer, Biolin Scientific, Gothenburg, Sweden). The crystals used, coated with SiO_2_ and with a resonance frequency of 4.95 MHz, were also purchased from Biolin Scientific. Prior to the measurement, the crystals were cleaned in an ultrasonic bath with chloroform, isopropanol, and water, each for 15 min and etched using the RCA-cleaning procedure (5:1:1 of water:NH_3_:H_2_O_2_ at 75 ${}^{\circ }C$ for 20 min). The baseline was recorded with the rinsing solution; the dipping order of the PE solutions was the same as in the corresponding dip-coating procedure. The time of adsorption and rinsing for each layer was the time until the frequency reached a steady state. The evaluation of the results was only done on the basis of the change in frequency and dissipation. The decrease in frequency was correlated with an increase in mass. An increase in dissipation indicates softening of the film. Comparing the different overtones of the signal gave additional information about the viscoelasticity of the thin film. Diverging overtones imply a flexible and soft film.

## 3. Results

First, the PEMs are characterized dependent on the preparation parameters NoBL, pH-value, and the PE concentration by AFM and ellipsometry. In addition, in situ QCM-D measurements are carried out, and the change in frequency (correlating with adsorbed mass) and dissipation (correlating with viscoelasticity) is determined. Afterwards, the swellability of PEMs is determined dependent on the varied parameters by measuring the change in thickness and refractive index with varying relative humidity.

### 3.1. PEMs’ Formation

**Figure 2:**
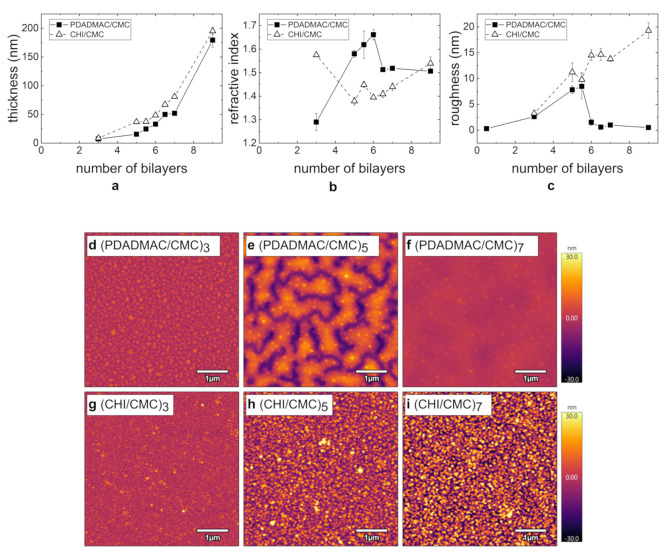
Summary of the morphology results for PEMs PDADMAC/CMC (filled squares) and CHI/CMC (empty triangles) from ellipsometry (**a**,**b**) and AFM measurements (**c**–**i**): (**a**) Film thickness, (**b**) refractive index, and (**c**) roughness with an increasing number of bilayers (NoBL). (**d**–**i**) AFM images (5 × 5 µm^2^) for PDADMAC/CMC and CHI/CMC at 3, 5, and 7 BL. For all images, the height scale is set to 60 nm. The samples were prepared at pH 4 and c${}_{PE}$ = 1 g/L. All experiments were carried out at RH ≈ 40%. The first bilayer corresponds to the bilayer of the precoat of polyethylenimine (PEI) and CMC.

PEMs prepared from the polycations PDADMAC and CHI (pH = 4; c${}_{PE}$ = 1 g/L) with a varying NoBL are characterized with respect to their thickness and morphology by ellipsometry (Figure 2a,b) and AFM (Figure 2c–i), respectively. In the figure, integers correspond to a bilayer terminating with the polyanion CMC and half numbers to the polycation as the outermost layer. The film thickness of both PE systems grows exponentially, whereas the CHI/CMC system results in slightly larger film thicknesses (Figure 2a). The refractive index grows with increasing film thickness and NoBL, with the exception at three BL, at which a much larger value is obtained (Figure 2b). In contrast, the PDADMAC/CMC system shows a significant increase in the refractive index with increasing NoBL. At six BL, a plateau is reached, leading to a constant value at higher BL. At nine BL, the refractive indices of both PE systems are similar to each other. A refractive index above 1.6 is uncommon for PEMs and is discussed in Section 4.1.

AFM measurements were carried out to determine the topography and the surface roughness of the PEMs. For a selected NoBL, AFM images of PDADMAC/CMC and CHI/CMC are shown in Figure 2d–i. In the case of PDADMAC/CMC at three BL (Figure 2d), an overall homogeneous PEM is obtained, which contains agglomerates at a size of a few tens of nanometers. At five BL (Figure 2e), pronounced domains form, and at seven BL (Figure 2f), a closed and homogeneous film is obtained. The comparison of the measured thickness and the height scale of the topography shows that the sample surface at five BL is not completely covered by the PEM. The roughness (Figure 2c) exhibits a maximum at 5.5 BL and then reaches a constant low value in the subnanometer range for PDADMAC/CMC. The comparison of the PEMs PDADMAC/CMC (Figure 2d) and CHI/CMC (Figure 2g) at three BL reveals that in both cases, a homogeneous surface is obtained, in the sense that there are no large agglomerates. With an increasing number of adsorbed bilayers, the surface roughness of CHI/CMC increases while the surface stays macroscopically homogeneous. Beyond six BL, CHI/CMC exhibits a significantly higher roughness than PDADMAC/CMC.

### 3.2. Adsorption Kinetics

**Figure 3:**
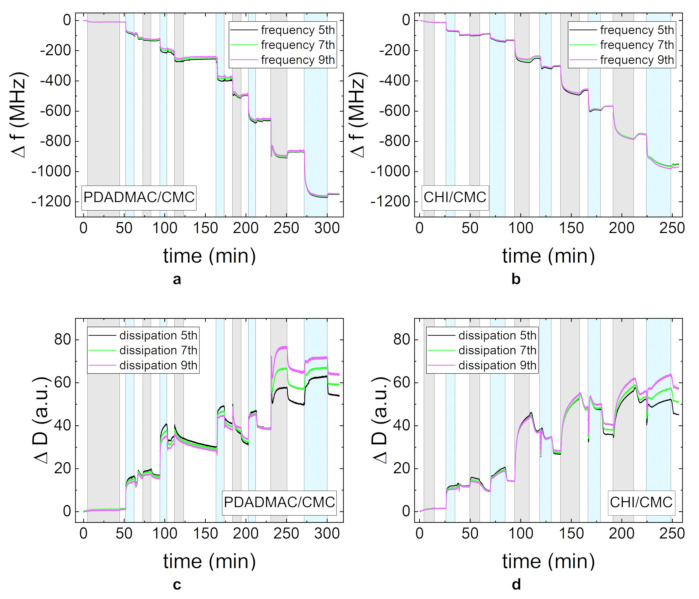
The change in frequency Δf (**a**,**b**) and dissipation ΔD (**c**,**d**) measured by quartz crystal microbalance experiments with dissipation monitoring (QCM-D) for the PEMs PDADMAC/CMC and CHI/CMC. The gray region is the time period in which the polycation is adsorbed and the blue region in which the polyanion is adsorbed. The different curves in one plot represent different overtones of the measured intensity (5th, 7th, and 9th). All PE concentrations were set to 1 g/L, and the pH-value was maintained at pH = 4.

To gain further insight into the adsorption behavior and to resolve the adsorption kinetics of the PEs, in situ QCM-D measurements were carried out. Figure 3 shows the change in frequency and dissipation for PDADMAC/CMC and CHI/CMC. To facilitate the interpretation of the data, the adsorption time periods are highlighted by different colors: gray for the adsorption of the polycation, blue for the polyanion, and white for the rinsing steps. The first area corresponds to the adsorption of the precursor layer PEI. Each adsorption step was carried out until the steady state was reached so that different adsorption times resulted. In the adsorption experiments of both PE systems, the overall frequency decreases, while the dissipation increases. The different overtones diverge with an increasing NoBL. Each adsorption step can clearly be distinguished, as the contact of the charged layer with the PE solution induces an immediate change in frequency and dissipation. The time and the progress of frequency and dissipation until the steady state is reached varies for the two PEMs and for the different PEs in one system. It can also be observed that the amount of adsorbed mass and the time until steady state generally increase with each bilayer. The rinsing steps lead at most to a small amount of desorption and a small decrease in dissipation.

Figure 3a shows an increase in mass for each of the adsorption steps of CMC in the PDADMAC/CMC PEM system. The dissipation first increases significantly and then reaches a steady state value (Figure 3c). The adsorption of CMC on the CHI layers follows a similar behavior in the first adsorption steps for the change in frequency and dissipation. For a higher NoBLs, the change in dissipation during the adsorption of CMC exhibits a sharp decrease after the initial increase (Figure 3d). The adsorption of CMC has a slightly higher impact on the change of both the frequency and the dissipation on the PDADMAC/CMC PEM than on the CHI/CMC PEM. The adsorption behavior of PDADMAC in the PDADMAC/CMC PEM differs noticeably from the expected growth behavior for the first layers. Both the frequency and the dissipation decrease during adsorption. At a higher NoBL, the adsorption returns to the expected decrease in frequency and increase in dissipation. The adsorption of CHI in the CHI/CMC PEM also shows an increase in frequency and a decrease in dissipation throughout each of the adsorption steps.

### 3.3. Influence of pH-Value

**Figure 4:**
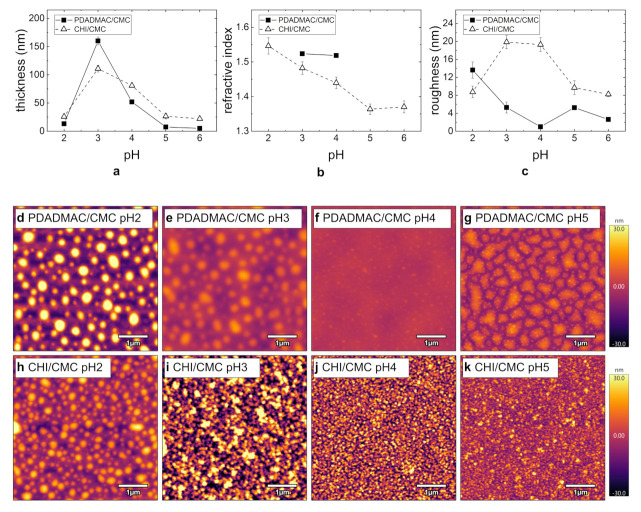
Summary of the morphology results for PEMS PDADMAC/CMC (filled squares) and CHI/CMC (empty triangles) from ellipsometry (**a**,**b**) and AFM measurements (**c**–**k**): (**a**) Film thickness, (**b**) refractive index, and (**c**) roughness with varying pH-values. (**d**–**k**) AFM images (5 × 5 µm^2^) for PDADMAC/CMC and CHI/CMC at pH = 2, 3, 4, and 5. For all images, the height scale is set to 60 nm. The samples were prepared at c${}_{PE}$ = 1 g/L and seven BL. All experiments were carried out at RH ≈ 40%.

The respective thicknesses and refractive indices measured by ellipsometry for the PEMs (seven BL; c${}_{PE}$ = 1 g/L) are shown in Figure 4. Both PDADMAC/CMC and CHI/CMC PEMs exhibit maximum film thickness of the film at pH 3 (Figure 4a). At pH 3, the film thickness of the PDADMAC/CMC PEM is larger than that for the CHI/CMC PEM, which is in contrast to all other pH-values. The refractive index decreases with increasing pH for the CHI/CMC PEM, reaching a constant value at about pH 5 (Figure 4b). For PDADMAC/CMC, only the refractive indices for pH 3 and pH 4 are shown. Due to the small film thickness, the fitting of independent parameters (thickness and refractive index) was not reliable for the other pH-values. Nevertheless, it can be observed that the refractive indices of the PEMs at pH = 3 and pH = 4 for PDADMAC/CMC are similar.

The topography of PDADMAC/CMC and CHI/CMC PEMs dependent on the pH and at seven BL was studied by AFM (Figure 4d–k). Height images reveal for both PE systems a strong agglomeration at pH 2 (Figure 4d,h). The agglomerates are spherical and have lateral diameters of several hundreds of nanometers. The height and lateral scale reveal that PDADMAC/CMC leads to larger agglomerates in all dimensions, but a lower packing density compared to the CHI/CMC PEM. With increasing pH, the PDADMAC/CMC agglomerates decrease in size, and the PEMs are overall smoother. The spherical aggregation progresses into a domain formation (Figure 4g). Figure 4c confirms the maximum roughness for pH 2 for PDADMAC/CMC, and the smoothest PEM with the lowest roughness is obtained at pH 4. This is in agreement with the topography images, as large agglomerates correlate with a high roughness. For the CHI/CMC PEM, the aggregate size decreases, and a higher packing density with increasing pH-value is obtained (Figure 4h–k). Here, the CHI/CMC PEMs have the largest roughness at pH 3 and pH 4 (Figure 4c). Overall, the roughness is generally lower for the PDADMAC/CMC PEMs compared to the CHI/CMC PEMs.

### 3.4. Influence of PE Concentration

**Figure 5:**
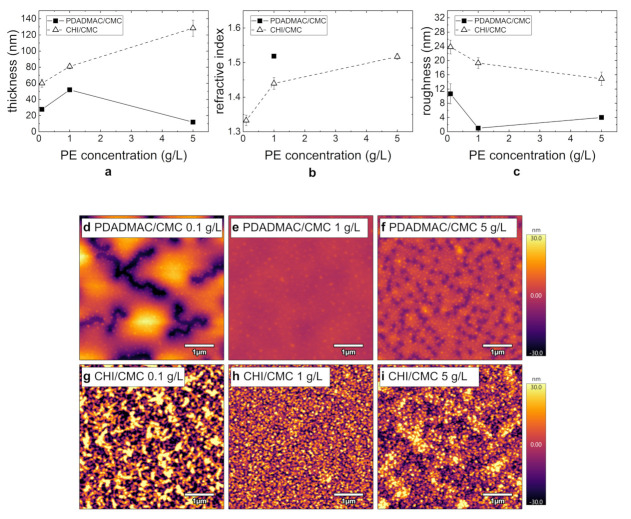
(**a**,**b**) Change in thickness and refractive index for PDADMAC/CMC (filled squares) and CHI/CMC (empty triangles) with varying PE concentrations determined by ellipsometry (RH ≈ 40%). (**c**) Change in roughness, determined by AFM (RH ≈ 40%), with varying PE concentrations for PDADMAC/CMC (filled squares) and CHI/CMC (empty triangles). (**d**–**i**) AFM images (5 × 5 µm^2^) for PDADMAC/CMC and CHI/CMC with varying PE concentrations. For all images, the height scale is set to 60 nm. The NoBL was set to seven, and the concentration of all solutions was set to 1 g/L. The first bilayer corresponds to the bilayer of the precoat PEI and CMC.

In the following, the influence of the PE concentration (0.1–5 g/L) on the thin film morphology is investigated. The respective morphology results of the prepared PEMs PDADMAC/CMC and CHI/CMC (seven BL; pH = 4) are summarized in Figure 5 as a function of the PE concentration. While the thickness of the PDADMAC/CMC PEMs is largest at a PE concentration of 1 g/L, the thickness of the CHI/CMC PEMs linearly increases with the PE concentration within the error bars (Figure 5a). For this system, the refractive index also increases with a rising PE concentration (Figure 5b). For the PDADMAC/CMC PEMs, the refractive index could only be determined for the thickest film prepared at 1 g/L.

The PEM topography with varying PE concentrations measured by AFM is shown in Figure 5d–i. While the PDADMAC/CMC PEMs prepared at 0.1 and 5 g/L partially dewet the substrate (Figure 5d,f), the PEM at 1 g/L is homogeneous (Figure 5e). The domain formation at 0.1 g/L is more prominent than at 5 g/L. In agreement with the topography results, the roughness for this system is lowest at 1 g/L and highest at 0.1 g/L. The surface of the CHI/CMC PEMs is more inhomogeneous for the PEMs prepared at 0.1 g/L (Figure 5g) and 5 g/L (Figure 5i) compared to the PEM prepared at 1 g/L (Figure 5h). The roughness decreases slightly with increasing concentration, which is in agreement with the topography images. Overall, the roughness of all CHI/CMC PEMs is significantly higher compared to the roughness of the PDADMAC/CMC PEMs.

**Figure 6:**
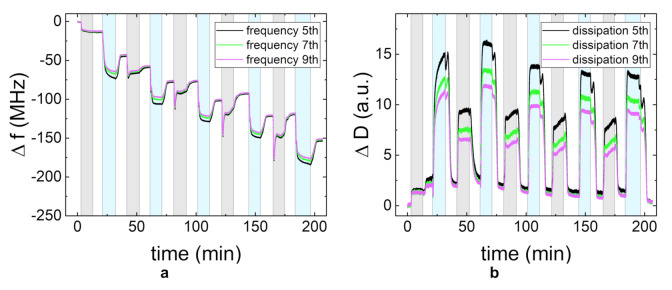
The change in frequency Δf (**a**) and dissipation ΔD (**b**) measured by QCM-D for the PEM PDADMAC/CMC at 5 g/L. The gray region is the time period during which the polycation is adsorbed, the blue region in which the polyanion is adsorbed, and white for the rinsing periods. The different curves in one plot represent different overtones of the measured intensity (5th, 7th, and 9th). The pH-value was maintained at pH = 4.

Since the low thickness of the PDADMAC/CMC PEM at 5 g/L (Figure 5a) was unexpected, the layer formation was additionally studied by QCM-D experiments (Figure 6). As the higher viscosity of the PE solutions impacts the shearing of the quartz crystal, a quantitative comparison between the different PE concentrations can only be done for the rinsing periods (Figure 6, white regions). The frequency change of the different rinsing periods shows a rather linear (instead of exponential) growth of the PEM. The decrease in frequency during the adsorption periods of CMC shows an increase in adsorbed mass (Figure 6a, blue regions). In contrast to this, the adsorption of PDADMAC (Figure 6a, gray regions) leads to an initial increase in adsorbed mass, followed by slow increase in frequency, thus a desorption. The rinsing process after each PDADMAC adsorption step results in a final frequency equal to the frequency prior to the adsorption step. This means that the adsorption and rinsing of PDADMAC has no impact on the overall change in frequency for this layer and with that on the total adsorbed mass on the substrate. In addition, the resulting frequency change is significantly lower than for the PEMs prepared at 1 g/L (Figure 3a,c). Figure 6b shows that the film viscoelastic properties do not change significantly, as the dissipation only increases by a much slower rate, if at all, compared to the previously studied systems (Figure 3b,d). The relatively stronger spreading of the overtones with respect to the overall dissipation during the adsorption steps is most likely an impact of the higher viscosity of the 5 g/L. The QCM-D measurements are in good agreement with the observed inhibited growth of the PDADMAC/CMC PEM prepared at 5 g/L (Figure 5a).

### 3.5. Swelling of PEMs

PEMs are responsive materials, which respond to the surrounding relative humidity with a thickness change of the thin film. The swelling behavior of the PEMs is studied by measuring the thickness with ellipsometry at varying relative humidity (0–90% RH). Through the increase of the relative humidity in the measuring chamber, the water content inside the PEM increases, leading to a swelling of the PEM. The swelling coefficient *S* (Equation (1)), being a measure of the water content, is calculated from the difference in thickness *d* and the thickness of the dry PEM $d_{0}$ (1% RH).

(1) $$S=\frac{d-d_{0}}{d}.$$

**Figure 7:**
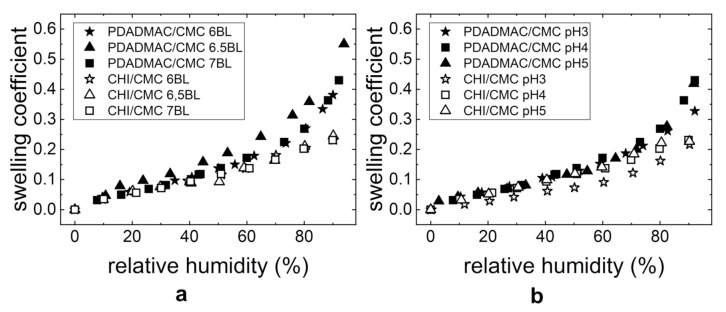
The swelling coefficient *S* dependent on the relative humidity RH of the PEMs PDADMAC/CMC and CHI/CMC with (**a**) varying NoBL (at pH = 4) and (**b**) different pH-values (seven BL). The thicknesses were measured by ellipsometry.

Figure 7 shows the swelling coefficient as a function of RH for both investigated PE systems. With increasing humidity, the film thicknesses of the studied PEMs PDADMAC/CMC and CHI/CMC increases (Figure 7a). Nevertheless, a different swelling behavior is observed for the two PE systems. The PDADMAC/CMC PEMs first swell linearly up to 70% RH. Above 70% RH, the film thickness swells exponentially. In addition, the water uptake depends on the kind of outermost PE layer (polycation or polyanion). As shown in Figure 7, the progression of the curves for the PEMs with CMC as the outermost layer (six BL and seven BL) is identical. For the PEM with PDADMAC as the outermost layer (6.5 BL), a higher water uptake is observed. In the case of CHI/CMC, the swelling coefficient increases linearly with the relative humidity and is independent of the outermost PE. When comparing both PE systems to each other, it can be seen that the PDADMAC/CMC PEMs have overall higher swelling ratios, thus incorporating a higher quantity of water than the CHI/CMC PEMs.

The swelling behavior was also studied for PEMs prepared at different pH-values, as the charge density most likely has a structural effect on the thin film. Figure 7b shows the swelling coefficient dependent on the relative humidity and pH. The PDADMAC/CMC PEMs swell in a similar manner at all pHs (pH 3, 4, and 5). The CHI/CMC PEMs at pH 4 and 5 show a similar linear swelling behavior compared to the PEMs presented in Figure 7a. The swelling behavior of the thin film prepared at pH 3 is lower than for the PEMs prepared at pH 4 and 5. An exponential upturn at around 70% RH leads to superposition with the PEMs prepared at pH 4 and 5.

## 4. Discussion

This work compares the influence of PE concentration, pH, and the NoBL on PEM formation composed of different polycations (PDADMAC or CHI) and a polyanion (CMC) with respect to the layer formation properties and surface topography characteristics. In addition, the swelling behavior at varying relative humidity is demonstrated for exemplary samples with a varying NoBL and different pH. The main analytical methods are AFM, ellipsometry, and QCM-D experiments.

In the following, first, the PEM formation process and the surface morphology are discussed. Next, the influence of the structure on the swellability of the PEMs is elaborated.

### 4.1. Multilayer Formation

Both PE systems (PDADMAC/CMC and CHI/CMC) grow exponentially with an increasing NoBL (Figure 2a and Figure 3a,c). An exponential thickness growth of the PEM is linked to a high mobility of the PEs [26]. The mobility of the PEs determines the ability for diffusion between the bulk PEM and the solvent. For example, PEs with a high charge density and no added salt, as is the case for PDADMAC (Figure 1c), do not diffuse into the PEM. Systems with immobile PEs, for example PDADMAC/PSS, lead to a linear thickness increase for each adsorption step [27,28]. From that, it is concluded that the exponential growth observed for the PDADMAC/CMC PEM results from a high mobility of CMC (Figure 1a). In the CHI/CMC system, both PEs are mobile enough in order to diffuse in the PEM. While the high mobility of CMC is deduced from the thickness results of the PDADMAC/CMC system, the mobility of CHI is already confirmed in the literature for the PE system hyaluronan/CHI [29]. Since the high mobility of the PE contributes to the thickness growth, it is expected that the mobile and coiled CHI (Figure 1b) contributes by a higher fraction to the thickness growth compared to the immobile and rather rigid PDADMAC molecules. This in turn means that the similar layer thicknesses (Figure 2a and Figure 3a,c) for both PE systems point to a synergistic effect of PDADMAC and CMC contributing to the large resulting thickness of the film. During the adsorption step of PDADMAC, CMC is assumed to diffuse out of the PEM bulk to the surface, leading to an intrinsic charge compensation [26,28,30,31]. This charge compensation in turn leads to a further PDADMAC adsorption. The pronounced adsorbed amount of PDADMAC is supported by a pronounced change in frequency (Figure 3a). Since PDADMAC is more rigid than CMC, a pronounced decrease in dissipation occurs at the same time. The complexation of CMC and PDADMAC at the PEM surface and the release of counter ions lead to a denser layer and reduced layer softness (or dissipation) [32].

The results of the pH variation on the PEM morphology are related to the PEM structure depending on the charge density of the PE (Figure 4). On the one hand, a high charge density correlates with a slower PE diffusion, and the adsorption of the stretched chains contributes less to the film thickness. On the other hand, a too low charge density can inhibit the formation of the PEMs. This is due to lacking a change in surface charge during the alternating adsorption of polycation and polyanion [33,34]. This becomes visible in the ellipsometry measurements (Figure 4a). PDADMAC is a strong PE carrying one permanent and pH-independent charge on each monomer (Figure 1c). CMC is a weak PE (Figure 1a, DS = 0.6, pK${}_{a}\approx$ 4), whose charge density varies with the protonation state and thus with the pH of the PE solution. At low pH-values, the charge density of CMC is very low due to protonation of the carboxylic group. This results in an inhibited PEM assembly, lower film thicknesses (Figure 4a), and higher agglomeration (Figure 4c). At pH 5 and 6, the higher charge density leads to the stretched chain conformation and small film thicknesses of the PEMs. At a pH-value between three and four, a balance between the charge density and the coiling of the chains exists, resulting in the observed maximum film thickness (Figure 4a). The effect of the PE solution pH on the film thickness is similar for the CHI/CMC as for the PDADMAC/CMC PEMs. This trend confirms the major effect of CMC on the pH dependency of the PEMs. The charge density of CHI (DS = 0.75, pK${}_{a}\approx$ 6.5) does not vary between pH 2 and 6 due to almost complete protonation over the entire pH range. A pH dependent effect of CHI is therefore not expected (see also Zhang et al. [24]) and was not observed during the experiments. At pH 3, a significantly higher film thickness is obtained for the PDADMAC/CMC PEM compared to the CHI/CMC PEM (Figure 4a) due to the higher charge density of PDADMAC than for CHI. Here, it is assumed that the mobility of the weakly charged CMC is further enhanced by PDADMAC than by CHI and a higher amount of PDADMAC is adsorbed.

The effect of the PE concentration of the dipping solutions is shown in Figure 5 and Figure 6. The formation of PEMs is strongly controlled by the adsorption rate of the PE to the surface of the PEM. Therefore, a decrease in PE concentration influences the amount of adsorbed PE [33]. If the PE concentration is lowered, but the adsorption time is maintained constant at 10 min, a reduced film thickness is expected [35,36,37]. This effect is confirmed for both PEM systems PDADMAC/CMC and CHI/CMC (Figure 5a). Here, the lowest PE concentration leads to the thinnest PEMs in both cases. The higher roughness observed (Figure 5c) may result from an insufficient surface coverage. As expected, an increasing PE concentration leads to a higher film thickness of the resulting PEMs (Figure 5a). For PDADMAC/CMC, the PEM formation is not monotonous since a stripping of PE complexes leads to partial desorption (Figure 5a and Figure 6). This effect was already observed by Sui et al. [38] and is confirmed by the results of this study.

### 4.2. Structure and Swellability

In this manuscript, it is demonstrated that the structure and morphology of the PEMs strongly depend on the preparation conditions such as the NoBL, pH, and PE concentration of the dipping solution. The domain formation shown in the AFM images (Figure 2d–f) is assumed to result from a lateral agglomeration during the drying of the PEM rather than by the rinsing steps [39,40]. The formation of large domains explains the high refractive indices (Figure 2b) and roughness (Figure 2c) of the intermediate NoBL. The high roughness and its potential influence on the optical fitting of the ellipsometric data can lead to an underestimated film thickness and overestimated refractive index [41,42]. The dewetting of the PDADMAC/CMC PEMs hints to a flexible thin film. This is further supported by the low local roughness (Figure 2c, after 6 BL), which is independent of the NoBL. The roughness is independent of the film thickness when the PEMs are flexible enough to decrease interfacial tensions, thus the roughness of the PEM, or for PEM systems with highly mobile PEs diffusing into the layer [40]. The high flexibility of the PDADMAC/CMC complexes facilitates its high water uptake (Figure 7a). The PDADMAC terminated PEM (Figure 7a, 6.5 BL) swells more strongly because of a higher concentration of counter ions [43] and therefore a higher osmotic pressure in the PEM [25,44]. This dependency of the water uptake on the outermost PE layer is commonly observed for PEMs and known as the odd-even effect [32,44,45,46,47].

The CHI/CMC system does not result in observable flexible PEMs. On the one hand, Figure 2c shows an increasing roughness with increasing NoBL, as commonly observed for PEMs [48]. This observation and the overall higher roughness compared to PDADMAC/CMC PEM indicates that the flexibility of the CHI/CMC PEM is not high enough in order to reduce interfacial tensions through a smoothing of the surface. On the other hand, the lower flexibility reduces the water uptake of the CHI/CMC PEMs (Figure 7). The overall lower water uptake of the CHI/CMC PEMs is assumed to result from a lower concentration of counter ions. Moreover, no prominent odd-even effect is observed since similar swelling coefficients are obtained for the PEM with CHI and with CMC as the outermost layer. However, the water uptake at pH 3 is lower compared to the PEMs prepared at the higher pH-values and increases exponentially above 60% RH. The strong chain coiling and low charge density of CMC at pH 3 seems to lead to a denser film with less counter ions present in the film. The non-linear behavior beyond 60% RH may result from an enhanced flexibility of the film at high humidity and the release of counter ions in the polymer coils. In conclusion, the higher flexibility of the PDADMAC/CMC PEMs is assumed to result from a higher concentration of counter ions generally present in the PEM. On the one hand, the higher concentration of counter ions presumably stems from a higher extrinsic charge compensation caused by the highly charged PDADMAC. On the other hand, the high mobility of CMC and CHI leads to a complexation of the two PEs and a decrease in counter ion concentration.

## 5. Conclusions

**Figure 8:**
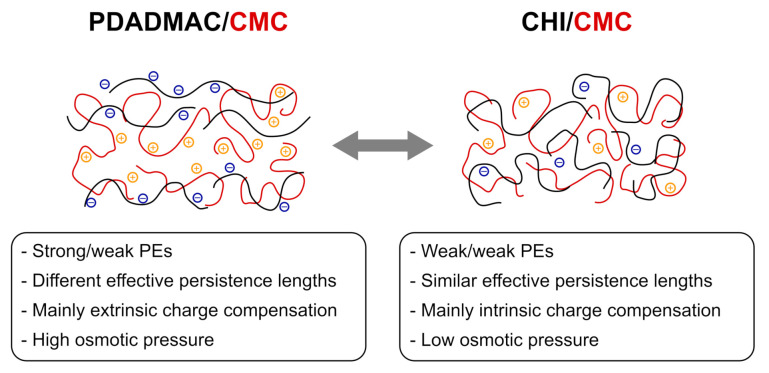
Proposed structures of the PEM systems PDADMAC/CMC and CHI/CMC, resulting from the high charge density of PDADMAC and the high effective persistence length. The polycations are depicted in black, the corresponding negatively charged counter ions in blue. The polyanions are depicted in red, the corresponding positively charged counter ions in orange.

In this work, thin films in the form of PEMs are prepared by alternating dip-coating of the cellulose derivate CMC and the positively charged polyelectrolytes PDADMAC or CHI. These PEMs enable the study of the interaction between cellulose fibers with functional additives used for the modification of paper products. By using the cellulose derivate CMC, the water insolubility of cellulose is overcome and the formation of PEMs enabled. These PEMs exhibit unique film properties such as a controllable thickness and surface topography, which were characterized with a broad variety of experimental methods. The study reveals that PEMs formed with PDADMAC and CMC are flexible thin films. The PEMs prepared with more than six BL have a low roughness and show a homogeneous surface topography, which can be related to highly charged PDADMAC chains, which are adsorbed in a more stretched conformation than the CMC chains. Figure 8 shows the proposed structure for both PEM systems. Due to the high charge density, PDADMAC (Figure 8, left) has a longer effective persistence length (including backbone rigidity and charge effects) than CMC and CHI. Therefore, 1:1 stoichiometric complexation between PDADMAC and CMC is not fulfilled, which leads to pronounced extrinsic charge compensation by non-adsorbed counter ions in the PEM. The high amount of counter ions leads to a high osmotic pressure and a high water uptake of the PDADMAC/CMC PEMs. In addition, a lower amount of complexation sites allows parts of the PEM to move, which might explain the ability to form lateral domains under certain conditions. The similarity of persistence lengths of CHI and CMC (originating from similar backbone and charge densities) leads mainly to intrinsic charge compensation and a low osmotic pressure of the CHI/CMC PEM (Figure 8, right). Since both polysaccharides have bulky monomers, they form quite large loops, leading to larger agglomerates (roughness) and thickness than the PDADMAC/CMC PEM under certain pH conditions.

Cellulose model surfaces are used for the study of the functionalization of paper, as it not only enables the broadening of the range of possible analysis methods, but also excludes errors from the randomness and complex structure of the fiber surface. The preparation of PDADMAC/CMC and CHI/CMC PEMs should allow an extensive study of fiber-polymer interaction. The PDADMAC/CMC PEMs are thin and smooth, independent of the NoBL (above a thickness of 50 nm). The smooth film enables the study of the chemical interaction of functional additives with the model surface. The surface of the CHI/CMC PEMs with controlled roughness can mimic the rough surface of a fiber and can be used to study the physical share of the interaction. It is known that the swelling of the fibers during paper preparation has an influence on the modification, and the studied swelling behavior of the PEMs can be used to further study the effect of the integration of the functional additives into the fiber wall on the functionalization. This in turn allows efficiently preparing cellulose based materials such as functional materials [49,50], filtering membranes [51], drug delivery systems [52], and bioassays [53].

## Figures and Tables

**Table 1:** Summary of the parameters for the two-layer model required for the analysis of the ellipsometric data. PEMs: polyelectrolyte multilayers.

Layer	Thickness (nm)	n	k
(humid) air	continuum	1.0000	0
PEMs	to be fitted	to be fitted	0
SiOx	1.1	1.4570	0
Si	continuum	3.8858	−0.0180

## Data Availability

All data used for evaluation in this manuscript are herein presented.
